# The hypoxia-inducible factor-1α activates ectopic production of fibroblast growth factor 23 in tumor-induced osteomalacia

**DOI:** 10.1038/boneres.2016.11

**Published:** 2016-07-05

**Authors:** Qian Zhang, Michele Doucet, Ryan E Tomlinson, Xiaobin Han, L Darryl Quarles, Michael T Collins, Thomas L Clemens

**Affiliations:** 1 Department of Orthopaedic Surgery, Johns Hopkins University, Baltimore, MD, USA; 2 Department of Medicine, University of Tennessee Health Science Center, Memphis, TN, USA; 3 Skeletal Clinical Studies Unit, Craniofacial and Skeletal Diseases Branch, National Institutes of Health, Bethesda, MD, USA; 4 Baltimore Veterans Administration Medical Center, Baltimore, MD, USA

## Abstract

Tumor-induced osteomalacia (TIO) is a rare paraneoplastic syndrome in which ectopic production of fibroblast growth factor 23 (FGF23) by non-malignant mesenchymal tumors causes phosphate wasting and bone fractures. Recent studies have implicated the hypoxia-inducible factor-1α (HIF-1α) in other phosphate wasting disorders caused by elevated FGF23, including X-linked hypophosphatemic rickets and autosomal dominant hypophosphatemia. Here we provide evidence that HIF-1α mediates aberrant FGF23 in TIO by transcriptionally activating its promoter. Immunohistochemical studies in phosphaturic mesenchymal tumors resected from patients with documented TIO showed that HIF-1α and FGF23 were co-localized in spindle-shaped cells adjacent to blood vessels. Cultured tumor tissue produced high levels of intact FGF23 and demonstrated increased expression of HIF-1α protein. Transfection of MC3T3-E1 and Saos-2 cells with a HIF-1α expression construct induced the activity of a FGF23 reporter construct. Prior treatment of tumor organ cultures with HIF-1α inhibitors decreased HIF-1α and FGF23 protein accumulation and inhibited HIF-1α-induced luciferase reporter activity in transfected cells. Chromatin immunoprecipitation assays confirmed binding to a HIF-1α consensus sequence within the proximal FGF23 promoter, which was eliminated by treatment with a HIF-1α inhibitor. These results show for the first time that HIF-1α is a direct transcriptional activator of FGF23 and suggest that upregulation of HIF-1α activity in TIO contributes to the aberrant FGF23 production in these patients.

## Introduction

Tumor-induced osteomalacia (TIO) is a rare, acquired paraneoplastic disorder characterized by a renal phosphate leak leading to hypophosphatemia and dysregulated bone turnover.^1–4^ Tumors associated with TIO are typically small, slowly growing neoplasms collectively referred to as phosphaturic mesenchymal tumor-mixed connective tissue variant.^5,6^ A significant proportion of these tumors are classified as hemangiopericytomas, but can also include sarcomas, ossifying fibromas, granulomas, giant cell tumors, and osteoblastoma.^5,6^ Patients with TIO present with bone pain, renal phosphate wasting, hypophosphatemia, low or inappropriately normal serum 1,25-dihydroxy vitamin D (1,25(OH)_2_ D) concentrations, and elevated serum alkaline phosphatase levels, all of which normalize upon tumor resection.^1–4^ The cause of the phosphate wasting is due to the aberrant secretion of fibroblast growth factor 23 (FGF23), a phosphate and vitamin D regulating hormone produced mainly by skeletal osteocytes, which is also known to cause other phosphate-wasting syndromes including autosomal dominant hypophosphatemic rickets (ADHR) and X-linked hypophosphatemic rickets.^7,8^

The precise molecular mechanism(s) that cause overproduction of FGF23 in phosphate wasting disorders have not been conclusively identified. However, recent studies have shown that patients with ADHR who carry mutations that disrupt the normal cleavage of intact FGF23 are predisposed to develop frank phosphate wasting if they are deficient in iron.^9^ These clinical observations and subsequent studies in a mouse model of ADHR^9^ suggested the involvement of the hypoxia-inducible factor-1α (HIF-1α), a transcription factor whose nuclear import and activity is regulated by via an iron-dependent prolyl hydroxylation reaction.^10^ Separate studies in infantile hemangiomas, which do not cause phosphate wasting but exhibit several characteristics, also evident in TIO, have demonstrated that increased HIF-1α-induced vascular endothelial growth factor (VEGF) receptor signaling promotes tumor progression by driving uncontrolled angiogenesis.^11,12^

On the basis of these two converging lines of evidence suggesting a role for HIF-1α in FGF23 production and in hemangioma tumor progression, we explored the hypothesis that aberrant FGF23 production in TIO tumors might also occur though a HIF-1α-dependent mechanism. Our results show that HIF-1α and FGF23 are coexpressed in tumors from patients with TIO and that HIF-1α is a direct transcriptional activator of FGF23. These findings suggest that upregulation of HIF-1α activity in this neoplasm contributes to the aberrant FGF23 production in TIO.

## Materials and methods

### Patients

Tumors from two patients with confirmed TIO were studied. Patient 1 was a 49-year-old female with longstanding bone pain and fractures. Preoperative biochemical workup showed a low serum phosphate (1.9 mg·dL^−1^; reference range: 2.5–4.5 mg·dL^−1^) and reduced 1,25-(OH)_2_D (19 pg·mL^−1^; reference range: 25–65 pg·mL^−1^) with elevated serum FGF23 (480 pg·mL^−1^; reference range <50 pg·mL^−1^). Patient 2 was a 54-year-old male who also presented with a history of bone pain and fracture. In this patient, preoperative serum phosphate was 1.8 mg·dL^−1^, 1,25-(OH)_2_D was 20 pg·mL^−1^ and FGF23 was elevated at 850 pg·mL^−1^. In both cases, the tumors were identified by a combination of functional (nuclear medicine studies) and anatomical imaging studies.^13^ Immediately after tumors were resected, serum phosphate levels returned to normal and intact FGF23 levels were undetectable, consistent with the resected tumor being the offending phosphaturic mesenchymal tumor (PMT) and cure.

### Tumor tissue culture and treatment

Freshly isolated tumor tissue was collected at surgery and promptly prepared for *in vitro* study. Tumors were finely minced with dissecting scissors and cultured for 24 h in Dulbecco's Modified Eagle's Medium containing 5% fetal bovine serum. In some cultures, digoxin (Sigma, St. Louis, MO, USA) was added at the beginning of the culture. Intact FGF23 concentrations were measured in conditioned medium using an ELISA kit (60–6100, Immutopics, San Clemente, CA, USA). Tumor tissue was collected for immunoblot analysis.

### Immunoblot analysis

Immunoblot was performed according to standard techniques. Primary antibodies (anti-HIF-1α (1:200, sc-10790, Santa Cruz Biotechnology, Santa Cruz, CA, USA) and anti-beta-actin (1:1 000, #3700, Cell Signaling Technology, Danvers, MA, USA) were incubated overnight at 4 °C.

### Cell culture and promoter analysis

MC3T3-E1 (ATCC CRL-2593, Rockville, MD) and Saos-2 (ATCC HTB-85, Rockville, MD) osteoblast cell lines and FGF23 reporter constructs were used for FGF23 promoter analysis, as previously described.^14^ Briefly, 3–5×10^4^ cells were seeded in 6 cm diameter tissue culture plates in αMEM media (Life Technologies, Grand Island, NY, USA) with 10% fetal calf serum at 37  °C in the presence of 5% CO_2_ in a humidified incubator. Cells were plated 18 h before transfection and fed fresh medium 4 h before transfection. FGF23 promoters (5ʹ mFGF-23 flanking region of the mouse 0.6 kb FGF23 promoter or 0.7 kb of the human FGF23 promoter) DNA were constructed into a *pGL3* basic reporter gene (Promega, Madison, WI, USA). FGF23 reporter plasmid DNAs were introduced into MC3T3-E1 or Saos-2 cells using cationic liposomes (LipofectAMINE2000, Life technologies). FGF23 promoter constructs (0.25 μg) were co-transfected with HIF-1α plasmid DNA (Novus Biologicals, Littleton, CO, USA) and assayed after 16–18 h. Cells were then washed twice with phosphate-buffered saline and incubated in fresh medium containing 10% fetal calf serum for 38 h. The HIF-1α activator L-mimosine (Sigma-Aldrich, St. Louis, MO, USA) or the HIF-1α inhibitor Bay87–2243 (Xcessbio, San Diego, CA, USA) was added to the cell cultures 24 h before the cells were collected. To standardize the transfection efficiency, 0.1 μg of pRL-CMV vector (pRL *Renilla reniformis* luciferase control reporter vector; Promega) was co-transfected in all experiments. Cells were collected 72 h after transfection and lysed in 50 μL of reporter lysis buffer (Promega). A luciferase assay (20 μL of cell lysed) was performed using a dual luciferase assay kit (Promega), and activity was measured with an Optocomp 1 luminometer (MGM Instruments, Hamden, CT, USA). Promoter activity (mean±s.d. of triplicate samples in relative fold changes) is represented by reporter activity normalized to pRL-CMV control.

### Chromatin immunoprecipitation assay

Chromatin immunoprecipitation (ChIP) assays were performed with a kit from Cell Signaling Technology according to the manufacturer’s instructions with the following modifications. Approximately 4×10^7^ MC3T3-E1 cells in 100 cm^2^ culture dishes were crosslinked in 1% formaldehyde solution (Fisher Scientific, Pittsburgh, PA, USA) for 10 min at room temperature; cross-linking was terminated by adding 1 mol glycine for 5 min at room temperature. Cells were washed twice with ice-cold phosphate-buffered saline (Life Technologies) and collected in 1 mL of phosphate-buffered saline+protease inhibitor cocktail+phenylmethylsulfonyl fluoride (Sigma-Aldrich). Cells were then pelleted by centrifugation at 1 500 r·min^−1^ for 5 min at 4 °C and the pellet was resuspended in 10 mL ice-cold buffer A with DL-dithiothreitol (DTT), protease inhibitor cocktail, and phenylmethylsulfonyl fluoride on ice for 10 min. Cell nuclei were pelleted by centrifugation at 3 000 r·min^−1^ for 5 min at 4 °C. Nuclei pellet was then washed in 10 mL ice-cold buffer B with DTT, and resuspended in 1.0 mL buffer B with DTT. Micrococcal nuclease was added to the nuclei and incubated for 20 min at 37 °C. Digestion was stopped by adding 100 μL of 0.5 mol·L^−1^ EDTA, and nuclei were pelleted by centrifugation at 13 000 r·min^−1^ for 1 min at 4 °C. The nuclear pellet was resuspended in 0.5 mL 1× ChIP buffer with protease inhibitor cocktail and phenylmethylsulfonyl fluoride for sonication using the VirSonic Ultrasonic cell disrupter 100 (VirTis, Gardiner, NY, USA) to shear the DNA to an average length of 300–500 base pairs (six 15 s bursts on ice). Samples were stored at −80 °C before use. For ChIP, 200 μL of the crosslinked chromatin preparation were added to 800 μL of ChIP buffer with protease inhibitor cocktail. Immunoprecipitations were carried out with 2 μg of antibodies (CREB, Est-1, or NFATc1 from Santa Cruz, CA, USA, and FGFR1 from Cell Signaling Technology) and 0.2 μg of an antibody against HIF-1α (Santa Cruz). Histone H3 rabbit mAb (2 μg) and normal rabbit IgG (1 μg) were used as positive or negative controls, respectively. After overnight immunoprecipitation on a rotator at 4 °C, ChIP samples were then washed and eluted and DNA was un-crosslinked with NaCl and subsequently treated with proteinase K, Tris-HCl and EDTA. DNA was purified and subjected to PCR amplification of FGF23 promoter DNA containing putative binding sites for HIF-1α transcription factors using specific primers (forward primer 5ʹ- TTTCAGTACTGCTGGCTGCC-3ʹ, reverse primer 5ʹ- TGCCGCCACATCCTCTGTGT-3ʹ).

### Histology and immunohistochemistry

Paraffin-embedded samples were cut into 5 μm sections and prepared for hematoxylin and eosin staining or immunohistochemistry as described previously.^15^ Briefly, after antigen retrieval, sections were treated using a CSA kit (K1500, DAKO, Carpinteria, CA, USA), then incubated in primary antibodies against HIF-1α (1:10 000, NB100-105, Novus, Littleton, CO, USA) and FGF23 (a gift from Immutopics) at 4 ˚C overnight. After incubation with biotinylated tyramide, sections were incubated with Alexa Fluor 488-linked Streptavidin (S-11223, Thermo Fisher Scientific, Waltham, MA) and 594-linked anti-goat antibody (A-11058, Thermo Fisher Scientific, Waltham, MA). Images were obtained using a fluorescence microscope (IX71 Olympus, Tokyo, Japan).

### Statistical analysis

All values are presented as mean±s.e.m. Differences between groups were analyzed either by the Student *t*-test or one-way analysis of variance followed by the Student–Newman–Keuls test, in which *P*<0.05 was considered statistically significant.

### Ethics statement

All clinical data and tissues were obtained from patients with informed written consent. Protocols for this study were approved by the Institutional Review Board of the National Institutes of Health. The investigation conformed to the principles outlined in the Declaration of Helsinki Principles.

## Results and discussion

**Figure 1:**
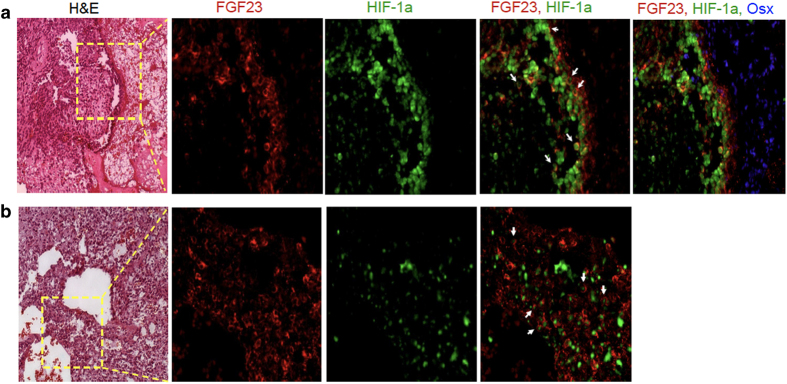
Colocalization of HIF-1α and FGF23 in tumors from patients with TIO. (**a,** **b**) Serial five micron sections from paraffin-embedded tumor tissue stained with H&E or processed for immunohistochemistry using fluorescent tagged antibodies as indicated. In both tumors, abundant FGF23 (red) was detected in the spindle-shaped cells adjacent to blood vessels and was observed to colocalize (white arrows) with HIF-1α immunoreactivity (green). Osterix immunoreactivity (blue) was observed in populations of cells adjacent but distinct from those expressing FGF23 and HIF-1α (**a**, far right). FGF23, fibroblast growth factor 23; H&E, hematoxylin and eosin; HIF-1α, hypoxia-inducible factor-1α; TIO, tumor-induced osteomalacia.

The main histopathological features of both tumors studied here are compatible with their classification as hemangiomapericytomas. These included extensive vascularization by vessels of variable diameter with neoplastic spindle-shaped cells adjacent to the endothelial layer (Figure 1a and b, far left panels). Both tumors exhibited regions of calcified matrix deposition containing chondroosteogenic-like cells, features typical of this type of tumor.^5,6^ Normalization of the biochemical and bone parameters after resection confirmed the nature of the tumor.

To begin to explore the possible link between HIF-1α and FGF23 in TIO, we performed immunohistochemistry in paraffin sections using well-characterized antibodies for each protein. HIF-1α and FGF23 immunoreactivity was co-localized to spindle-shaped cells adjacent to blood vessels (Figure 1a and b). By contrast, osterix positive cells were generally not observed to colocalize with HIF-1α and FGF23 but instead were detected in the regions of matrix deposition (Figure 1a, far right panel).

**Figure 2:**
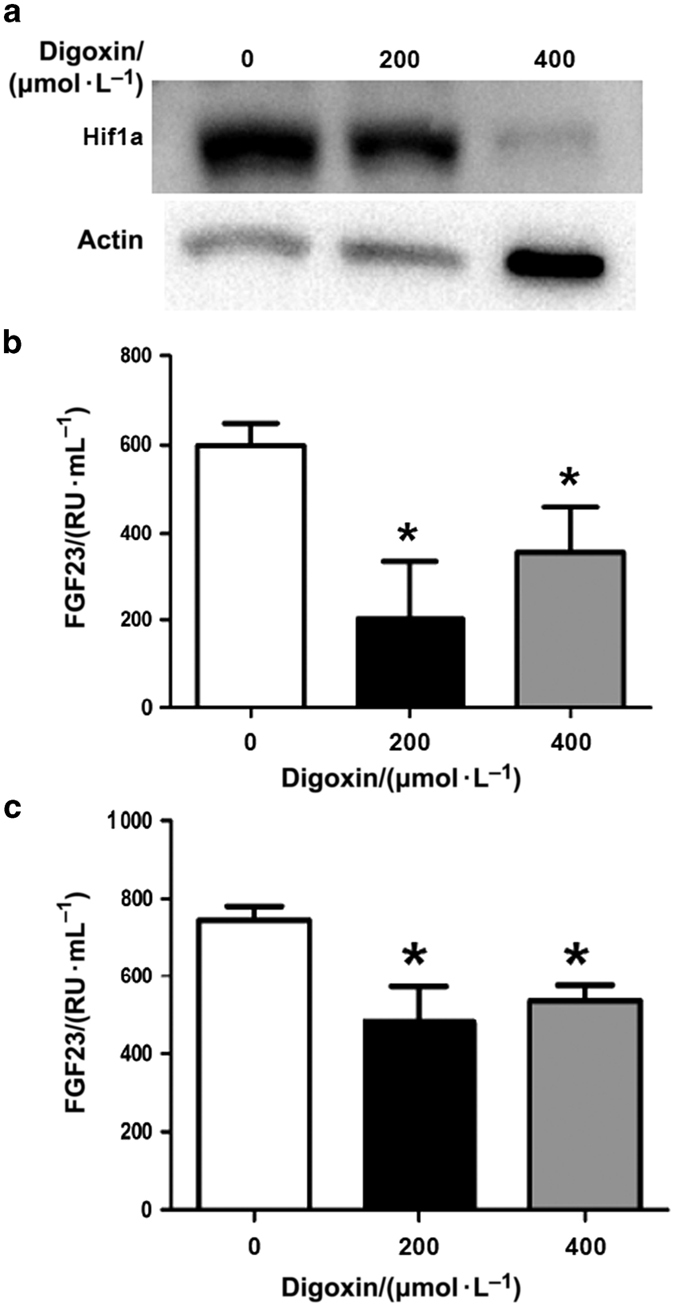
HIF-1α expression in tumors from patients with TIO is inhibited by treatment with digoxin. Tumors resected from patients with proven TIO were cultured *in vitro* and HIF-1α protein was measured before and after treatment with digoxin. (**a**) HIF-1α protein expression analyzed by immunoblotting of cultured tumor tissue in the presence or absence of 200 or 400 μmol·L^−1^ digoxin. (**b**, **c**) Intact Immunoreactive FGF23 concentrations measured in conditioned medium from two different tumors grown in organ culture in the absence (0) or presence of 200 or 400 μmol·L^−1^ digoxin. FGF23, fibroblast growth factor 23; HIF-1α, hypoxia-inducible factor-1α; TIO, tumor-induced osteomalacia. *P<0.05.

To investigate the role of HIF-1α on ectopic FGF23 production in TIO, we performed immunoblot analysis and measured FGF23 levels in conditioned medium from tumor tissue cultured for 24 h in the presence or the absence of digoxin, which inhibits the translation of HIF-1α mRNA.^16,17^ In untreated tumor tissue, HIF-1α protein expression was readily detected by immunoblotting (Figure 2a), whereas mRNA expression was unchanged (data not shown) consistent with the well-established posttranscriptional regulation of this transcription factor.^10^ FGF23 protein levels in medium were increased within 1 h (data not shown) and remained elevated throughout the culture period (Figure 1b and c). Treatment with digoxin decreased HIF-1α protein and reduced FGF23 protein levels in culture medium from both tumors (Figure 2b and c).

**Figure 3:**
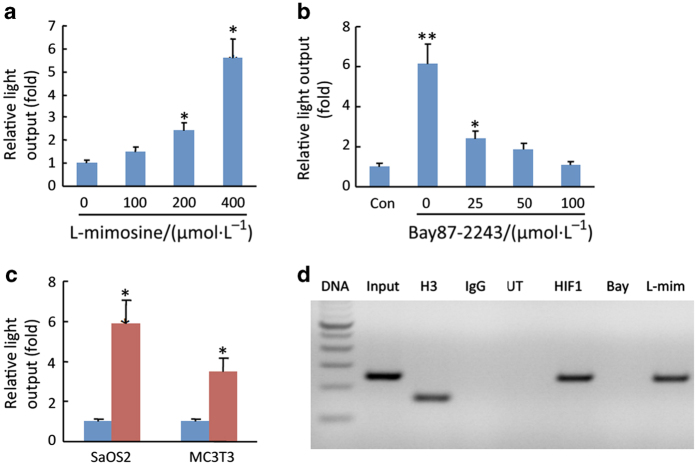
Regulation of *FGF23* gene expression by HIF-1α. (**a**) FGF23 promoter activity in Saos-2 and MC3T3-E1 osteoblasts co-transfected with a HIF-1α DNA (0.25 μg). (**b**) Effect of L-mimosine on HIF-1α, induced FGF23 promoter activity in MC3T3-E1 osteoblasts. (**c**) Effect of Bay87–2243 on HIF-1α induced FGF23 promoter luciferase activity in MC3T3-E1 osteoblasts. (**d**) ChIP analysis of HIF-1α occupancy at endogenous consensus elements within the FGF23 promoter in MC3T3-E1 cells. Lane 1. 100 bp DAN ladder; Lane 2. Input ChIP DAN; Lane 3. H3-positive control; Lane 4. Non-specific IgG; Lane 5. Untreated control; Lane 6. Co-transfection with HIF-1α; Lane 7. Extracts from Bay87–2243 treated cells showing inhibition of HIF-1α binding to the endogenous FGF23 promoter; Lane 8. Extracts from L-mimosine treated cells showing increased HIF-1α binding to the endogenous FGF23 promoter. Data are expressed as the mean±standard error (*n*=3) from three separate experiments. FGF23, fibroblast growth factor 23; HIF-1α, hypoxia-inducible factor-1α. *P<0.05, **P<0.005.

To determine whether HIF-1α directly regulated FGF23 transcription, we examined FGF23 promoter luciferase activity in osteoblasts *in vitro* following the induction of HIF-1α. FGF23 promoter activity was increased in a dose-dependent fashion by the iron chelator L-mimosine (Figure 3a). The increased promoter luciferase activity in L-mimosine-treated cells was inhibited by pretreatment with the HIF-1α inhibitor Bay87–2243 (Figure 3b). For these experiments, we used Bay87–2243 instead of digoxin because of its lower cellular toxicity under these experimental conditions. Forced expression of HIF-1α in both Saos-2 and MC3T3-E1 cells co-transfected with pcDNA3-HIF-1α significantly increased FGF23 luciferase activity compared with those transfected with the pcDNA3 empty vector (Figure 3c). To determine whether HIF-1α increased FGF23 through direct promoter binding, we performed ChIP assays using a HIF-1α antibody. ChIP analysis revealed HIF-1α binding to a consensus HIF-1α binding site in the proximal FGF23 promoter, which was eliminated in cells treated with Bay87–2243 (Figure 3d). This finding defines *cis* elements in the proximal FGF23 promoter that are necessary for *FGF23* gene transcription. This finding, together with the fact that HIF-1α is upregulated in FGF23 producing cells in these tumors, strongly suggests that HIF-1α is a central mediator of ectopic FGF23 production in TIO.

The studies reported herein focus on HIF-1α in the pathogenesis of TIO and were prompted by separate but converging lines of evidence supporting this concept. As mentioned above, studies in a mouse model of ADHR confirmed the role of iron deficiency in elevating FGF23, and parallel *in vitro* studies demonstrated that iron chelators increased FGF23 in association with elevated HIF-1α protein^9^ suggesting that upregulation of HIF-1α in iron deficiency might activate *FGF23* gene transcription and thereby predisposes patients with ADHR to manifest hypophosphatemia. Our demonstration that HIF-1α is a potent transcriptional activator of FGF23 supports this concept. A second line of inquiry has implicated increased HIF-1α activity in the vascular abnormalities associated with progression of other endothelial tumors. Thus, Medici and Olsen^11^ demonstrated that the hyperproliferation of vascular endothelial cells in cutaneous infantile hemangiomas depended on HIF-1α-mediated VEGF receptor signaling. Such studies suggest that upregulation of HIF-1α in the setting of these endothelial neoplasms is secondary to the genetic mutations that initiates and promotes endothelial tumor progression. In the case of TIO, the marked upregulation of HIF-1α would appear to also lie downstream of a primary mutation in these tumors. In this regard, recent studies have identified a gene rearrangement in a significant proportion of tumors causing TIO, which results in fibronectin 1(FN1)/FGFR1 fusion protein capable of FGFR signaling.^18^ Unfortunately, we were not able to determine whether this rearrangement was present in the tumors from the patients described here due to lack of sufficient tumor material for analysis. Nonetheless, our findings link HIF-1α and FGFR signaling in the pathogenesis of TIO and suggest feasible approaches for better diagnosis and treatment of this paraneoplastic syndrome.
